# Differences in Looking at Own- and Other-Race Faces Are Subtle and Analysis-Dependent: An Account of Discrepant Reports

**DOI:** 10.1371/journal.pone.0148253

**Published:** 2016-02-05

**Authors:** Joseph Arizpe, Dwight J. Kravitz, Vincent Walsh, Galit Yovel, Chris I. Baker

**Affiliations:** 1 Laboratory of Brain and Cognition, National Institute of Mental Health, National Institutes of Health, Bethesda, Maryland, United States of America; 2 Applied Cognitive Neuroscience Group, Institute of Cognitive Neuroscience, University College London, London, United Kingdom; 3 Department of Neurology, University of Tennessee Health Science Center, Memphis, Tennessee, United States of America; 4 Le Bonheur Children’s Hospital, Memphis, Tennessee, United States of America; 5 Department of Psychology, The George Washington University, Washington, D.C., United States of America; 6 Department of Psychology, Tel Aviv University, Tel Aviv, Israel; Bournemouth University, UNITED KINGDOM

## Abstract

The Other-Race Effect (ORE) is the robust and well-established finding that people are generally poorer at facial recognition of individuals of another race than of their own race. Over the past four decades, much research has focused on the ORE because understanding this phenomenon is expected to elucidate fundamental face processing mechanisms and the influence of experience on such mechanisms. Several recent studies of the ORE in which the eye-movements of participants viewing own- and other-race faces were tracked have, however, reported highly conflicting results regarding the presence or absence of differential patterns of eye-movements to own- versus other-race faces. This discrepancy, of course, leads to conflicting theoretical interpretations of the perceptual basis for the ORE. Here we investigate fixation patterns to own- versus other-race (African and Chinese) faces for Caucasian participants using different analysis methods. While we detect statistically significant, though subtle, differences in fixation pattern using an Area of Interest (AOI) approach, we fail to detect significant differences when applying a spatial density map approach. Though there were no significant differences in the spatial density maps, the qualitative patterns matched the results from the AOI analyses reflecting how, in certain contexts, Area of Interest (AOI) analyses can be more sensitive in detecting the differential fixation patterns than spatial density analyses, due to spatial pooling of data with AOIs. AOI analyses, however, also come with the limitation of requiring a priori specification. These findings provide evidence that the conflicting reports in the prior literature may be at least partially accounted for by the differences in the statistical sensitivity associated with the different analysis methods employed across studies. Overall, our results suggest that detection of differences in eye-movement patterns can be analysis-dependent and rests on the assumptions inherent in the given analysis.

## Introduction

Fixations are thought to reflect the allocation of visual attention to specific features of the world and eye movements appear to be functionally useful in visual recognition [1,2]. As such, analysis of eye fixations can potentially provide insight into cognitive processing and, in particular, the stimulus features used in a given task. Thus, eye-tracking is a useful technique to study the basis of the Other-Race Effect (ORE) which is the robust and well-established finding that people are generally poorer at facial recognition of individuals of another race than of one’s own race [3–14]. In particular, eye-tracking can potentially provide insight into the long unresolved questions of which specific facial features are attended during own- and other-race face processing and whether there are differences in the diagnostic value of facial features among races. Surprisingly, although the ORE has been studied for over forty years and the eye-tracking technique has been used to study face perception for even longer [15], to date only a handful of research groups have studied the ORE utilizing eye-tracking [16–32].

While such eye tracking studies have provided valuable data for advancing our understanding of the basis of the ORE, they present highly conflicting results from which two competing views have emerged with respect to own- and other-race face identification mechanisms. The first view is that the fixation pattern to faces depends on the culture of the observer, but is equivalent for different races of faces [16–25]. The bulk of the evidence for this first view comes from Caldara and colleagues, employing variations on a paradigm and highly similar analysis techniques across studies. The second view is that the fixation pattern to faces does not depend on the race of observer but does differ for own- vs. other-race faces[26,32]. In accord with this second view, three recent studies [27–29,31] provide further evidence that fixation patterns differ between races of faces, though they did not test differences between races of observers. In light of these conflicting reports, the aim of the current study was to address the fundamental question of whether eye-movement patterns from a given observer differ for same- versus other-race face identification and to understand the potential basis for the conflicting reports.

Although the eye-tracking studies described above utilize different paradigms (e.g. 2, 5, and 10 second encoding, different pre-stimulus fixation locations, etc), stimuli (neutral vs. emotional faces, frontal vs. half-profile, Asian vs. African faces, etc), eye tracking measures (relative frequencies vs. durations, etc), and analyses (maps vs. Areas of Interest, etc), it is surprising that such fundamentally different results and interpretations have emerged among studies with essentially the same task, namely to encode and recognize same- and other-race faces.

To investigate eye-tracking patterns to own- and other-race faces, bridge the gaps between prior studies, and to investigate a factor that may at least partially account for the disagreement among prior results, we built several features into the design and analysis of our study. First, we used two other-race face categories (African and Chinese), in contrast to prior studies, which typically only used one. Second, we employed multiple analytic approaches, including the use of both AOI (Area of Interest) and heatmap analyses. Third, we controlled and systematically varied the fixation location prior to stimulus onset to detect differences in fixations to other race faces independent of visuomotor factors [33,34]. Finally, we obtained eye-tracking data during both the encoding and recognition phases of the study to test for replication of results.

We found that for Caucasian observers, recognition for Chinese faces was impaired relative to Caucasian and African faces. Further we found evidence for significantly increased fixations to the eyes in own- (Caucasian) versus other-race (African and Chinese) faces, and significantly increased fixations to the mouth for other- than own-race faces. Compared to Caucasian faces, Chinese faces also elicited significantly more fixations in the nose region. Thus, we conclude that other-race faces elicit different fixation patterns compared to own-race faces. These differences between face races were relatively small, however, thus potentially explaining the conflicting reports in prior studies regarding the influence of race of face on eye-movement patterns. We suggest that differences in paradigm and limitations of the analyses in prior studies may have led to a lack of sensitivity for this effect in some studies [16–19,23–25], and a detection of this effect in others [26–29,31].

## Methods

### Ethics Statement

All participants gave written informed consent and were compensated for their participation. The study was approved by the Institutional Review Board of the National Institutes of Health, Bethesda, Maryland, USA.

### Participants

30 Caucasian participants (11 male), living in the Washington D.C. area. One participant’s data was excluded from analyses of test phase eye-movements due to partial data corruption.

### Eye-tracking

We used an EyeLink II headmounted eye-tracker (SR Research, Mississauga, ON, Canada), and sampled pupil centroid at 500 Hz. The default nine point calibration and validation sequences were repeated throughout the experiment. Both eyes were calibrated and validated, but only the eye with the lowest maximum error was recorded for the trials following a particular calibration. Calibration was repeated when maximum error at validation was more than 1° of visual angle. Before each trial, a drift correction was performed. Default criteria for fixations, blinks, and saccades as implemented in the Eyelink system were used.

### Stimuli

**Fig 1:**
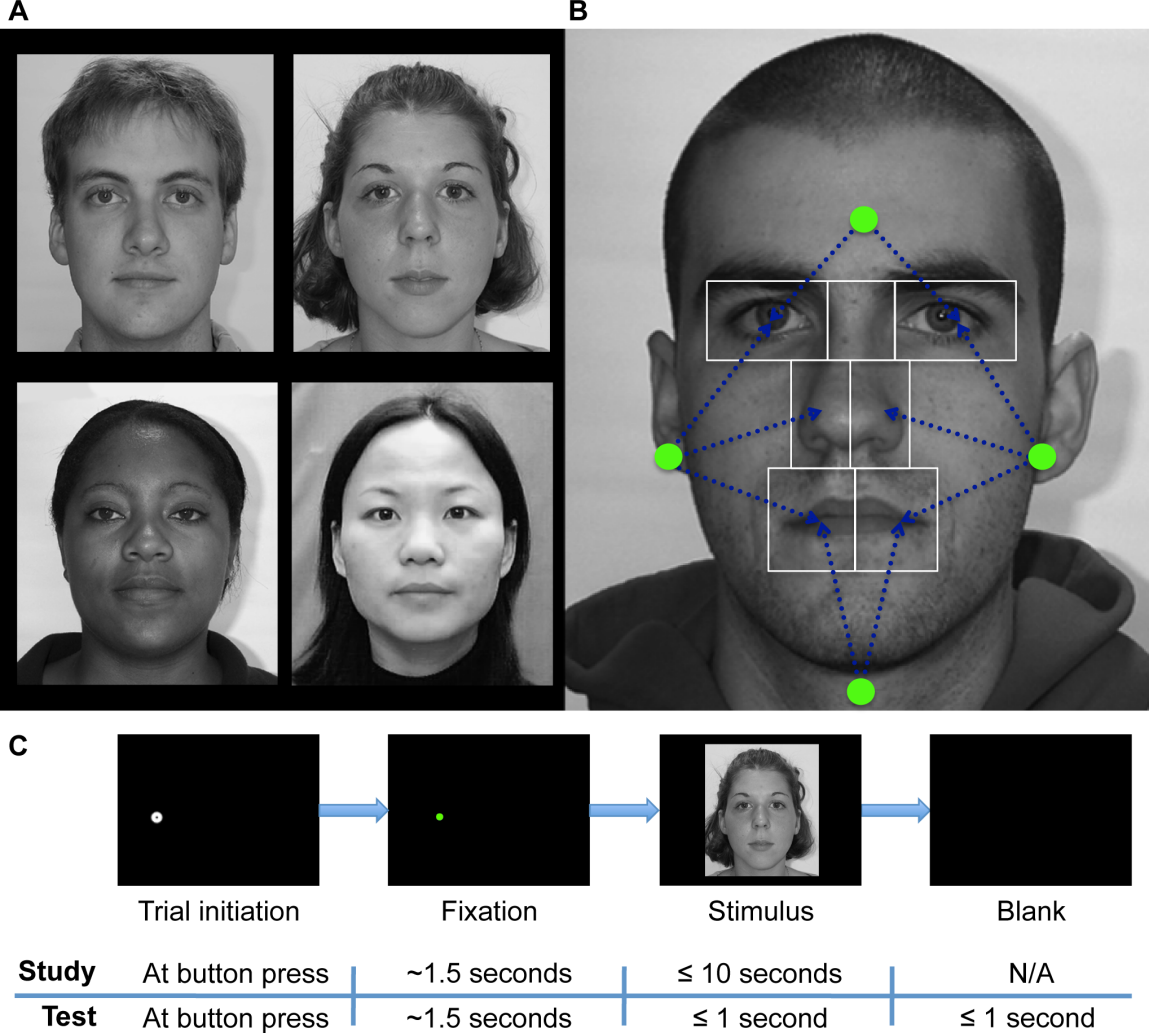
Study design. (a) Four example face stimuli. (b) AOIs for one face showing the calculation of start positions, which were determined separately for each face and defined relative to that face. Green dots schematically illustrate the potential start positions relative to the upcoming face. Left and right start positions were equidistant from centers of the nearest eye, nose and mouth AOIs. Upper and lower start positions were equidistant from the centers of the two eye or two mouth AOIs, respectively. Dotted blue lines schematically illustrate that the start positions were equidistant from the centers of the indicated AOIs. (c) Trial sequences in study and test phases. A face was only presented if the participant successfully maintained fixation for a total of 1.5 seconds. After face onset in the study phase, participants were free to study the face for up to 10 seconds and pressed a button to begin the next trial. In the test phase, faces were presented for one second only and participants responded with button presses to indicate whether the face was ‘old’ or ‘new’.

We collected 32 Caucasian-American, 32 African-American, and 32 Chinese face images (16 male and 16 female for each race), for a total of 96 grayscale neutral expression frontal-view face images (see Fig 1A for examples). All Caucasian faces were taken from the neutral expression 18 to 29 age group of the Productive Aging Lab Face Database established by the University of Texas at Dallas (http://vitallongevity.utdallas.edu/stimuli/facedb/categories/neutral-faces.html) [35]. African-American faces were taken from the neutral expression 18 to 29 age group of the Productive Aging Lab Face Database, from the MacBrain (“NimStim”) Face Stimulus Set made by the MacArthur Foundation Research Network on Early Experience and Brain Development (http://www.macbrain.org/resources.htm), and from the Color FERET Database (http://www.nist.gov/itl/iad/ig/colorferet.cfm) [36,37] established by the United States Department of Defense (DOD) Counterdrug Technology Program. All Chinese faces were taken from the CAS-PEAL Face Database (http://www.jdl.ac.cn/peal/index.html) [38] established by the ICT-ISVISION Joint Research and Development Laboratory (JDL) for Face Recognition. The use of different face databases for different races of faces meant that not all aspects of the images were uniform, and this could spuriously impact results. Therefore in order to address this limitation, the images were modified to make several image properties alike across all face stimuli. Each face was scaled to have a forehead width subtending 10 degrees of visual angle at presentation and was rotated to correct for any tilt of the head. Images were cropped to remove most of the background, but not the hair or other external features, and all images were equated for overall luminance. At presentation, images were centered on a black background. To eliminate any possible stimulus bias as the source of any laterality effects, half of the faces were randomly left-right flipped across the vertical midline of the image for each participant.

The website of the Productive Aging Lab Face Database states: “This [database] contains a range of face of all ages which are suitable for use as stimuli in face processing studies. Releases have been signed by the participants we photographed and the faces may be included in publications or in media events.” Development of the MacBrain Face Stimulus Set was overseen by Nim Tottenham and supported by the John D. and Catherine T. MacArthur Foundation Research Network on Early Experience and Brain Development. Please contact Nim Tottenham at tott0006@tc.umn.edu for more information concerning the stimulus set. Portions of the research in this paper use the FERET database of facial images collected under the FERET program, sponsored by the DOD Counterdrug Technology Development Program Office. The research in this paper use the CAS-PEAL-R1 face database collected under the sponsor of the Chinese National Hi-Tech Program and ISVISION Tech. Co. Ltd.

### Areas of Interest (AOIs)

For the purposes of analysis and for aspects of our experimental design, rectangular areas-of-interest (AOIs) were manually drawn prior to the experiment for each face around the right and left eyes, bridge of nose (i.e. middle of eye region), right and left half of nose, and right and left half of mouth (Fig 1B, for example) using EyeLink Data Viewer software. These AOIs were never visible to participants during the experiment.

The edges of the AOIs were defined with designated criteria that were applied to all face stimuli as consistently as possible with respect to the spacing relative to relevant facial feature landmarks. Specifically, eye AOI edges were defined in the x-dimension as just beyond each canthus of the eye, with slightly more space between the lateral canthi and the respective edges of the AOIs than that for the medial canthi due to the nearness of the bridge of the nose to the medial canthi. In the y-dimension, the eye AOI edges were defined as from the lower section of the malar fold to just beyond the superior eyelid. The bridge of nose AOIs were defined in the x-dimension as the whole space between the two eye AOIs. In the y-dimension, the edges were identical to those of the eye AOIs. The nose AOI was defined in the x-dimension as just beyond the edges of the alar lobules. In the y-dimension, the lower edge of the nose AOI bisected the philthrum and the top edge was identical to the lower edges of the eye and bridge AOIs. The mouth AOI was defined in the x-dimension as just beyond the oral commissures. In the y-dimension, the lower edge was at a similar position relative to bottom edge of the lip that tended to land about the labiomedial crease, when it was visible. The upper edge of the mouth AOI was identical to the lower edge of the nose AOI. When nose and mouth AOIs were split into two halves (for the purpose of placing the pre-stimulus start positions), simple bisection in the x-dimension was performed.

We subsequently conducted analyses of AOI areas, widths, and heights to provide rough indices of race of face physiognomic differences and variability (Figures F-H in S1 File).

### Design

We varied race of face stimulus (Caucasian, African, Chinese) and pre-stimulus fixation location (“start position”) across the trials of the experiment comprised of two phases: study and test. We systematically varied start position because fixation patterns are affected by visuo-motor factors (e.g. start position) in addition to stimulus factors (face) [33,34]. During the study phase, participants observed 48 faces (16 of each race, 8 male for each race) in a self-paced manner (up to 10 seconds, self-terminating trials with a button press). At test, participants observed 96 faces (the 48 study phase faces plus 48 new faces) for a limited duration (one second only) and indicated whether or not they recognized each face as one observed during study (old/new task) with a button press. Participants were instructed to respond within two seconds following stimulus onset, as soon as they thought they knew the answer (Fig 1C). The experiment was programmed in Python and interfaced with the eye-tracker.

Start positions were either above, below, right of, or left of the internal features of the upcoming face stimulus (see Fig 1B for examples). Coordinates for a given start position were calculated uniquely for each face stimulus to be equidistant from all of the nearest internal facial features. Specifically, for right and left start positions, the unique coordinate that was equidistant from the centers of the nearest eye, nearest half-nose, and nearest half-mouth AOI was calculated numerically for each face. Upper start positions were equidistant from the center of the two eye AOIs, and the lower start positions were equidistant from the two half-mouth AOIs. Distances from the upper and lower start positions to their respective AOI centers were constrained to be the mean of the right and left start position distances from their respective AOI centers.

Before stimulus onset, participants fixated at the start position, indicated by a standard Eyelink II calibration target (0.17° diameter black circle overlaid on a 0.75° diameter white circle) on the black screen. Participants initiated the trial by pressing a button while looking at the fixation target. In this period, a drift correction was performed. A colored dot (0.5° diameter) remained after drift correction, and the stimulus appeared only after a participant had fixated at the dot for an accumulated total of 1500 ms. This ensured that drift correction and fixation were stable prior to stimulus onset. If more than 1500 ms of fixation away from the start position accumulated before the trial could be initiated, drift correction was repeated. A fixation was considered off the start position if it landed more than 0.5° from the center of the dot. Dot color changed successively from red to yellow to green in order to signal to the participant that a maintained fixation was successfully detected at the start position.

In both the study and test phases, there were equal proportions of trials for each combination of levels of the factors of face race, face gender, and start position. When a given face was presented in both the study and test phases, the face images were identical across study and test phases. This practice had the advantage of making analysis more straightforward and easily interpreted since changes in viewpoint, emotional expression, lighting, etc. would not serve as confounds for eye-movement differences; however, this practice also has the limitation of potentially allowing simple image matching mechanisms, in addition to the more abstract facial identification mechanisms of interest. The particular subset of faces used in the study phase was randomized across participants. Of the faces presented in both study and test phase, half of the faces were presented with the same start position at study and test and for the other half, the start position on the other side of the face was used (e.g. left to right start position between study and test; upper to lower between study and test).

### Analyses

#### Software

Fixation and AOI data were obtained through EyeLink Data Viewer software by SR Research. Subsequent analyses on these data and behavioral data from the test phase were performed with custom Matlab (The MathWorks, Inc., Natick, MA, USA) code. ANOVAs were performed in SPSS (IBM, Somers, NY).

#### Behavior

We assessed participants’ discrimination performance, response bias, and reaction time on the old/new recognition task in the test phase. d' (d’ = z(hit rate)—z(false alarm rate)) and criterion c (c = -[z(hit rate) + z(false alarm rate)]/2) were computed for discrimination performance for each participant, broken down by race of face and start position. Reaction times were analyzed for correct trials only. Reaction time values more extreme than 2.5 standard deviations from the median within each condition and participant were excluded from analysis. Reaction time analyses were broken down by Race of Face and Start Position conditions with analysis being performed on the medians calculated for each subject. Greenhouse-Geisser correction was applied if any of the factors or interactions of a given ANOVA violated sphericity.

#### AOI Analyses

We assessed the relative frequencies of fixations across the AOIs as a function of our experimental manipulations. The AOIs used were left eye, bridge, right eye, nose (left and right sides combined to be comparable with prior studies), mouth (left and right sides combined), and other (outside the defined AOI regions). Given the variable numbers of fixations across trials and across participants, only the first five fixations of each trial were included in the analyses of the study phase. Participants rarely made fewer than five fixations in study phase trials and, further, the first few fixations are likely to be the most essential for the task, as indicated in prior research [39]. For the test analysis phase, all fixations within the entire stimulus viewing time (limit of one second per trial) were included. Relative frequency was calculated for each AOI as the number of actual fixations divided by the total number of possible fixations across all trials of the given condition for each subject (e.g. 16 study phase trials with Chinese faces multiplied by 4 fixations per trial = 64 total possible fixations across all Chinese face study phase trials). ANOVAs on relative frequencies excluded the relative frequency value for the region outside of the AOIs. Greenhouse-Geisser correction was also applied if any of the factors or interactions of a given ANOVA violated sphericity

#### Spatial Density Analyses

We mapped the spatial density of fixations during the study phase as a function of our experimental manipulations. Each fixation was plotted with equal density and spatial extent, and fixations were not weighted by the fixation duration (essentially the same qualitative pattern of results was obtained when this weighting function was applied). Fixations beyond the fifth fixation were excluded from the analysis to ensure an equal amount of data across trials. The first fixation was also excluded (See Results for motivation). To ensure that summation of fixation maps across different face trials produced spatially meaningful density maps, fixation maps for individual faces were first aligned to a common reference frame using simple translations only. This reference frame was defined by the internal facial features. Specifically, the alignment minimized the sum of the squared differences between the center of the AOIs for each face and the average centers of the AOIs across all 96 faces. Within this common reference frame, fixations were then plotted as Gaussian densities with a mean of 0 and a standard deviation of 0.3° of visual angle in both the x and y dimensions. These density plots were then averaged across trials and across participants. A small proportion of analyzed fixations (< 2% during study, < 1% during test) fell outside of the bounds of the stimulus image region (i.e. onto the black background outside the square frame of the face stimulus). These fixations were not excluded from the analyses, but are simply not visible in plots. The resulting maps show the spatial fixation densities, using a color scale from zero to the maximum density value observed, with values approaching zero being deep blue. All maps within a single figure contain the same total number of fixations and so are scaled the same to allow for direct comparison.

#### Spatial Density Contrasts: Difference Maps

In order to view differences in the spatial fixation density between two conditions, a pixel-wise subtraction between two spatial density maps was performed for each participant and then averaged across participants.

#### Spatial Density Contrasts: Statistical Maps

In order to produce maps of statistically significant differences in the spatial density map contrasts, a Monte Carlo permutation test was performed on fixation locations between the contrasted conditions. A Monte Carlo permutation test (also called an approximate permutation test or a random permutation test) is a standard, accurate and robust method of performing a significance test on data that is not known to have a parametric (e.g. normal) distribution of values, such as our data. We have used this type of statistical analysis method on eye-tracking data in a previous study [34], based on methods applied to the analysis of functional brain imaging data [40] and similar to that used in a prior study of eye tracking [41].

The null hypothesis in the Monte Carlo permutation tests was that the distributions of fixation locations of each ordinal fixation (i.e. fixation 2, fixation 3 etc.) were the same between the contrasted conditions (e.g. fixation 2 in Caucasian versus Chinese trials, or fixation 3 in right start position versus left). Thus, exchangeability of fixation locations between the given contrasted conditions was assumed only for fixations of the same ordinal value in the sequence of five fixations per trial. Only the first five fixations were analyzed for the same reasons that only the first five fixations were analyzed in the AOI analyses. 104,000 resampling iterations were performed for each statistical map. For each iteration, locations of fixations were resampled for each individual participant according to the assumed exchangeability, then a new resampled spatial density contrast was produced. These resampled maps were then averaged across participants to produce 104,000 group difference maps, the distribution of which was used to determine significance. Maps of p-values were computed pixel-wise based on the number of corresponding pixels in the resampling iterations that were greater than a given positively valued pixel (i.e. where condition 1 had a greater density) in the true spatial density contrast and that were less than a given negatively valued pixel (i.e. condition 2 greater) in the true spatial density contrast. The maps were thresholded at a pixel significance of p < 0.01 (equivalent to two-tailed p < 0.02).

For eye-tracking data, our statistical analysis has advantages over other methods of performing significance tests on contrasted fixation maps. Statistical analysis upon contrasted fixation maps can present particular problems to which AOI analyses are much less susceptible due to the high degree of pooling of fixation data in AOI analysis and due to the much more limited number of statistical tests involved in AOI analysis. Specifically, a pixel-wise t-test on fixation maps is inappropriate because the within-subject differences in fixation density data often deviate extremely from a normal distribution at many pixels of a heatmap. Even pixel-wise non-parametric tests could create a large multiple comparisons problem, which grows as the pixel resolution of heatmaps grow. In our analysis, fixation locations are exchanged rather than pixels; therefore, increasing the resolution at which heatmaps are displayed does not exacerbate the multiple comparisons problem. Our analysis is an alternative to a another approach, which has been implemented by Caldara and colleagues in a free Matlab toolbox called iMap [42].

#### Spatial Density Contrasts: Correction for Multiple Comparisons on Statistical Maps

In order to reduce the chance of false positives in our statistical maps due to multiple comparisons, we utilized False Discovery Rate (FDR) control, which enables setting the statistical thresholds to those at which a given estimated rate of false positives can be attained. The AFNI (http://afni.nimh.nih.gov) function 3dFDR was applied to each of the statistical maps. Because approximately half of the pixels in the statistical maps did not correspond to face stimulus pixels and because our aim was to detect fixation differences over internal facial features, the same non-face region mask was applied to all statistical maps before FDR correction so that those pixels would be ignored in the 3dFDR algorithm. Our FDR threshold was set to q < 0.05, at which it would be estimated that 5% of surviving pixels are false positives. Cluster size correction is an alternative method to FDR control for multiple comparisons correction, though in the context of this study, where fine-grained mapping of highly significant regions is preferred to detection of larger area regions, we chose to employ FDR control.

#### Profile Density Analyses

Because AOI analyses can be criticized for requiring a highly subjective *a priori* segmentation of visual features [42], but spatial statistical maps can be criticized for lacking sensitivity, we conducted additional exploratory analyses that were meant to increase sensitivity without subjective segmentation. In particular, we calculated profile densities (i.e. densities summed along a single dimension of a heatmap) for the different conditions during the study phase. The y-profile plots were the result of summing along the horizontal dimension (x-axis) of a spatial density heatmap. The y-profile plots visualize fixation density over specific facial features without respect to laterality or fine differences in horizontal position. Since the primary effects of interest here focused on which facial features were fixated (eyes, nose, mouth), we report only y-profile plots.

#### Profile Density Contrasts: Difference Plots

In order to visualize potential differences in profile density between two conditions, spatial density difference maps were summed along the vertical dimension to produce x-profile density difference plots and summed along the horizontal dimension to produce y-profile density difference plots. X-profile density difference plots visualize potential differences in left-right face laterality between contrasted conditions, and y-profile density difference plots visualize potential differences in density over specific facial features.

#### Profile Density Contrasts: Profile Statistical Maps

To find regions of statistically significant difference in the profile density difference plots, we re-used the 104,000 resampled iterations from the spatial density contrast statistical map analyses to perform a Monte Carlo permutation test on the contrasted profile plots. All resampled iterations from the relevant spatial density Monte Carlo permutation test were summed along the vertical dimension to produce the resampled iterations of the x-profile Monte Carlo permutation test, and were summed along the horizontal dimension to produce the resampled iterations of the y-profile Monte Carlo permutation test. P-values were computed pixel-wise (i.e. at each pixel along the relevant dimension) based on the number of corresponding pixels in the resampling iterations that were greater than a given positively valued pixel (i.e. where condition 1 had a greater profile density) in the true profile density difference plot and that were less than a given negatively valued pixel (i.e. condition 2 greater) in the true profile density difference plot. Maps visualizing the results were thresholded at a pixel significance of p < 0.025 (equivalent to two-tailed p < 0.05). The threshold p-value for the uncorrected profile density maps was numerically higher than that for the uncorrected spatial density maps, in part to accommodate for the fewer multiple comparisons, but also for demonstrative purposes to qualitatively compare effects with those of the AOI analyses. In these maps, pixels along the entire orthogonal dimension were highlighted where the dimension of interest had a significantly different profile density between contrasted conditions.

#### Profile Density Contrasts: Correction for Multiple Comparisons on Profile Statistical Maps

FDR control with threshold of q < 0.05 was again employed, but this time utilizing all pixels for each profile statistical map.

## Results

### Discrimination, Criterion, and Reaction Time

#### Reduced Discrimination for Chinese Faces, Conservative Bias for Caucasian Faces

**Fig 2:**
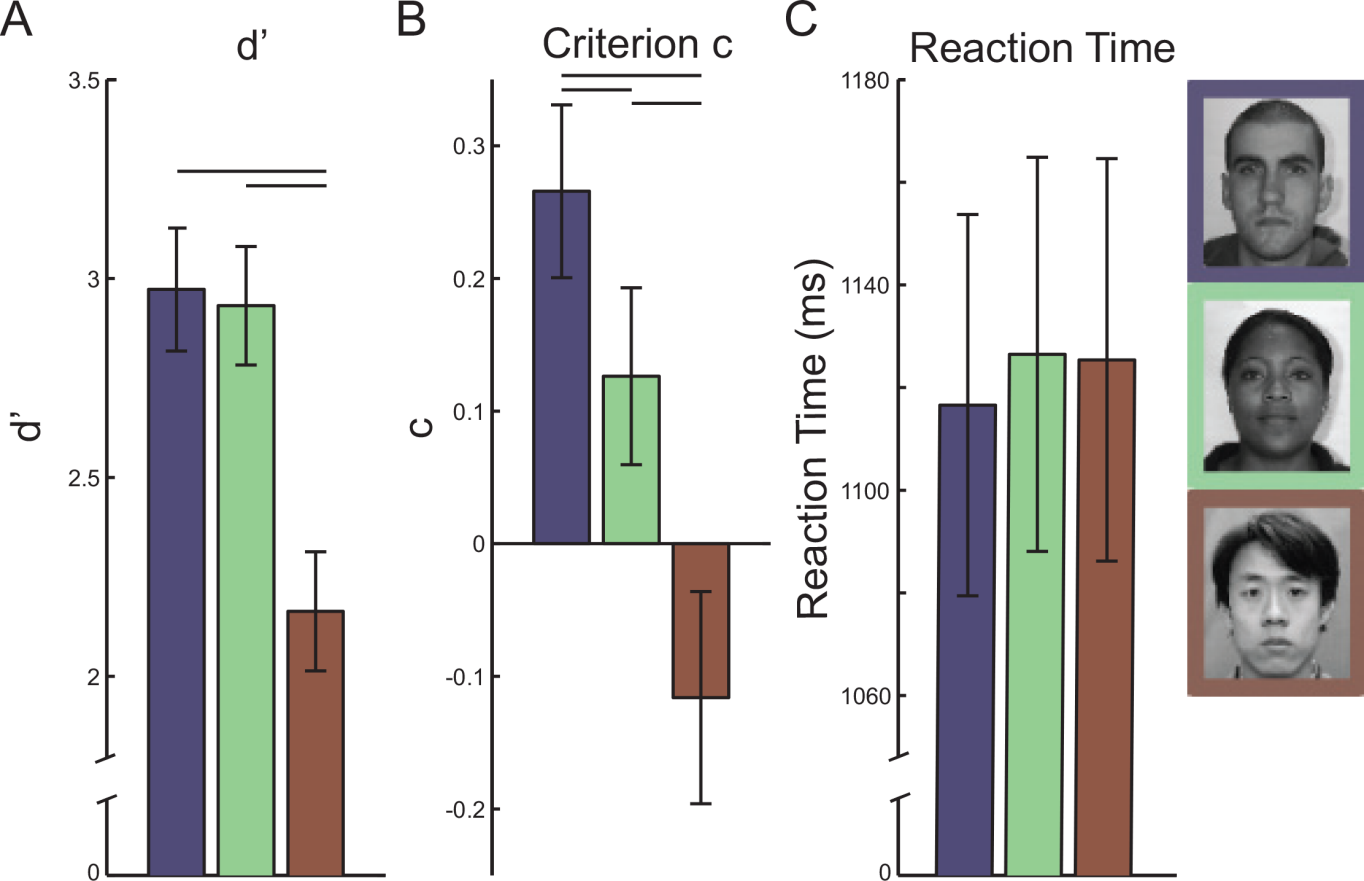
Effects of race of face on recognition performance. (a) Face recognition performance, measured by d’, was significantly lower for Chinese compared to Caucasian and African faces. (b) Criterion scores for Caucasian faces were higher (stricter) than African and Chinese faces. Also criterion scores for African faces were higher than for Chinese faces. (c) Reaction times did not differ among different race faces. Error bars indicate the between-subjects standard error.

Consistent with prior reports, we observed evidence for an Other-Race Effect in the discrimination scores (d’) and criterion scores (c) of our Caucasian participants, particularly for Chinese faces (Fig 2). A two-way ANOVA on discrimination scores (Fig 2A), with Race (Caucasian, African, Chinese) and Start Position (left, right, up, down) as within-subject factors revealed a significant main effect of Race (F(2,58) > 29.20, p < 0.001, η_p_^2^ = 0.50), but no main effect or interactions involving Start Position (p > 0.19, η_p_^2^ < 0.053). Pairwise Race comparisons, pooling across Start Position, revealed significantly better discrimination performance for Caucasian (t(29) > 5.99, p < 0.001, one-tailed, bias corrected G_Hedges_ = 0.94) and African (t(29) > 5.36, p < 0.001, two-tailed, bias corrected G_Hedges_ = 0.31) faces than Chinese faces. No significant difference was found between Caucasian and African discrimination (p > 0.33, one-tailed, bias corrected G_Hedges_ = 0.048). Thus an other-race discrimination deficit was observed for Chinese faces only.

Criterion (c) scores estimated bias in responding that a face was recognized, where a higher criterion score indicates a stricter criterion or more reluctance to respond that a face was recognized. A two-way ANOVA on criterion scores (Fig 2B), with Race and Start Position as within-subject factors revealed a significant main effect of Race (F(1.42,41.06) > 7.84, p < 0.005, Greenhouse-Geisser corrected, η_p_^2^ = 0.213), but no main effect or interactions involving Start Position (both p > 0.27, Greenhouse-Geisser corrected, η_p_^2^ < 0.044). Pairwise Race comparisons, pooling across Start Position, revealed significantly higher criterion scores for Caucasian faces than African faces (t(29) > 2.24, p < 0.017, one-tailed, bias corrected G_Hedges_ = 0.37) and Chinese faces (t(29) > 3.60, p < 0.0008, one-tailed, bias corrected G_Hedges_ = 0.93). Also, criterion scores were higher for African faces (t(29) > 2.39, p < 0.024, two-tailed, bias corrected G_Hedges_ = 0.58) than Chinese faces. One-sampled t-tests on criterion scores for each race revealed that only scores for Caucasian faces significantly differed from zero (Caucasian: p < 0.0005, two-tailed, Other Races: p > 0.072, two-tailed). Together these results reveal that other-race faces elicited a less conservative criterion to report that a face was recognized, with Chinese faces eliciting the least conservative.

There were no differences in viewing time in the study phase (mean study viewing time was 7023 ms (418 ms standard error) for Caucasian faces, 7112ms (450 ms standard error) for African faces, and 6933ms (415 ms standard error) for Chinese faces) or reaction time during the test phase (Fig 2C). A two-way ANOVA on study viewing time did not yield any significant main effects of Race or Start Position or any interaction (all p > 0.51). Similarly, a two-way ANOVA on reaction time data did not yield any significant main effects of Race or Start Position or any interaction (all p > 0.26).

### Temporal Dynamics of Fixations

In our previous studies of facial fixation patterns [33,34], we found that the first ordinal fixation was shorter in duration than subsequent fixations, suggesting that facial information for identification was not deeply processed during the first fixation (see also Hsiao & Cottrell, 2008), and focused our analyses on the subsequent fixations only. The same effect was observed in the current data (see S1 File Supplementary Material for full details), and so we excluded the first ordinal fixation in our analyses of the spatial distribution of fixations below, though qualitatively the same results were observed when it was included (reported in S1 Supplementary Material). Importantly, Race of Face did not significantly influence the temporal dynamics of eye-movements (Figures A and B in S1 File). Additionally though, we found an influence of Phase (Study, Test) on the temporal dynamics of fixations (Figure D in S1 File), which may reflect the influence of the time restriction in the test phase. Therefore, our main analyses focus on data from the Study Phase during which eye-movements were unrestricted.

### Spatial Patterns of Fixations

#### AOIs: Independent Influences of Race of Face and Start Position

**Fig 3:**
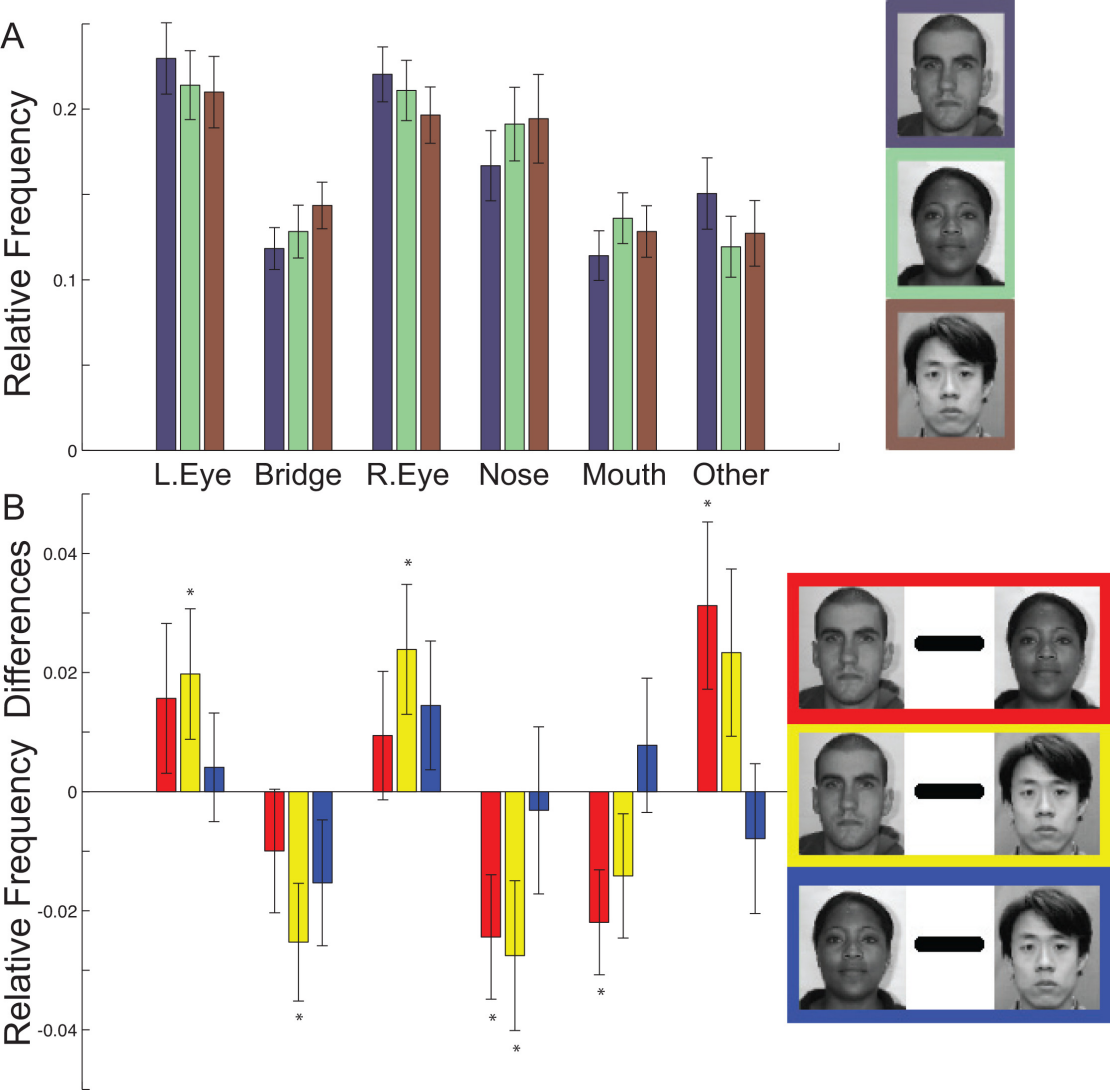
Distribution of fixations across AOIs for own- and other-race faces during the study phase. (a) Relative frequencies of fixations for each race of face across AOIs for the second through fifth fixations pooled. Error bars indicate between-subject standard errors. (b) Within-subject differences among race of face conditions from (a) reveal significantly more eye fixations for Caucasian than Chinese faces, significantly fewer nose fixations for own- than other-race faces, and significantly fewer mouth fixations for Caucasian than African faces. Error bars indicate the within-subject standard error.

We next focused on the specific pattern of fixations with the first fixation excluded (Fig 3). Area of Interest (AOI) analyses revealed that Race and Start Position both influence fixation patterns, but also indicate that their influences are independent. A three-way ANOVA on the relative frequency of fixations in the study phase with AOI (left eye, bridge, right eye, nose, and mouth), Race (Caucasian, African, Chinese) and Start Position (left, right, up, down) as within-subject factors, revealed a significant main effect of AOI (F(2.81,81.55) > 5.24, p < 0.004, Greenhouse-Geisser corrected, η_p_^2^ = 0.15), indicating that not all AOIs were fixated with equal frequency. There were no other main effects, neither for Start Position (F(2.78,80.61)>2.61, p>0.060, Greenhouse-Geisser corrected, η_p_^2^ = 0.083) nor for Race (F(1.96,56.78)>2.75, p>0.070, Greenhouse-Geisser corrected, η_p_^2^ = 0.087), but both of these factors interacted separately with AOI. Critically, the significant interaction between Race and AOI (F(6.31, 183.00) > 2.43, p < 0.025, Greenhouse-Geisser corrected, η_p_^2^ = 0.077) indicates that fixation patterns varied by Race. The significant interaction between Start Position and AOI (F(7.24, 209.84) > 17.51, p < 0.001, Greenhouse-Geisser corrected, η_p_^2^ = 0.38) replicates our previous work [33,34] demonstrating that fixation patterns vary by Start Position. There was no interaction between Start Position and Race (F(4.77, 138.40) < 0.79, p > 0.54, Greenhouse-Geisser corrected, η_p_^2^ = 0.027) and no significant three-way interaction among AOI, Race, and Start Position (F(12.60, 365.53) < 1.44, p > 0.14, Greenhouse-Geisser corrected, η_p_^2^ = 0.047), suggesting that the influences of Race and Start Position on fixation pattern are independent. The effect size of the AOI x Start Position interaction (η_p_^2^ = 0.38) was greater than that of the AOI x Race interaction (η_p_^2^ = 0.077), indicating that the independent effect of Start Position on the spatial pattern of eye-movements was greater than that of Race.

These patterns in the study phase were replicated in the test phase (Figure C in S1 File) in a similar three-way ANOVA on the relative frequency of fixations in the test phase with AOI, Race, and Start Position as within-subject factors. This revealed a significant main effect of AOI (F(2.26,63.31) > 4.026, p < 0.020, Greenhouse-Geisser corrected, η_p_^2^ = 0.13) in the test phase, again indicating that not all AOIs were fixated with equal frequency. Again, there was no main effect of Start Position (F(1.91,53.43)>1.48, p>0.23, Greenhouse-Geisser corrected, η_p_^2^ = 0.051), but there was a main effect of Race (F(1.95,54.45)>5.06, p<0.011, Greenhouse-Geisser corrected, η_p_^2^ = 0.15). Both of these factors interacted separately with AOI. The significant interaction between Race and AOI (F(5.89, 164.90) > 4.55, p < 0.0005, Greenhouse-Geisser corrected, η_p_^2^ = 0.14) again critically indicates that fixation patterns varied by Race. The significant interaction between Start Position and AOI (F(5.76, 161.40) > 13.06, p < 0.0005, Greenhouse-Geisser corrected, η_p_^2^ = 0.32) additionally replicates that fixation patterns vary by Start Position. There was no interaction between Start Position and Race (F(4.96, 138.92) < 1.17, p > 0.32, Greenhouse-Geisser corrected, η_p_^2^ = 0.040) and no significant three-way interaction among AOI, Race, and Start Position (F(12.37, 346.42) < 1.51, p > 0.11, Greenhouse-Geisser corrected, η_p_^2^ = 0.051), suggesting again that the influences of Race and Start Position on fixation pattern are independent. The effect size of the AOI x Start Position interaction (η_p_^2^ = 0.32) in the test phase was greater than that of the AOI x Race interaction (η_p_^2^ = 0.14), further indicating that the independent effect of Start Position on the spatial pattern of eye-movements was greater than that of Race. Given the independence of the effects of Start Position and Race and because the influence of Start Position has been thoroughly investigated in two previous studies [33,34], the following analyses will mainly focus on the influence of Race of face stimulus on eye-movements.

#### AOIs: Small but Systemic Influences of Race of Face Stimulus

The preceding analyses suggest an effect of Race of face on fixation patterns. Relative frequencies of fixations in our AOIs for Caucasian and Chinese faces (Fig 3A) highly resembles the analogous plot in a prior study (Fig 2 in Goldinger et al., 2009, which is the data for Caucasian observers looking at Caucasian and Asian faces with five second encoding duration), even though the precise way our AOIs were drawn differs slightly and our data utilizes only the second through fifth fixations. Notably, as previously indicated, relative frequency differences between own- and other-race faces (Fig 3A for study phase, Figure Ca in S1 File for test phase) in the left eye, right eye, nose, and mouth AOIs all, without exception, tended in the same direction as the prior study, specifically reflecting a relatively greater proportion of fixations over both eyes in own-race faces and relatively greater proportion of mouth and nose fixations for other-race faces. To test more rigorously for replication of this prior study [26], we conducted planned pairwise comparisons testing the hypotheses of relatively greater proportion of fixations for own- (Caucasian) versus other-race (African or Chinese) faces in each eye AOI and relatively greater proportion of fixations for other- versus own-race faces in the nose and mouth.

For the study phase (Fig 3), the tests for each of the two eye AOIs revealed significant differences in the hypothesized direction only for Caucasian versus Chinese faces (both eyes t(29) > 1.76, p < 0.044, one-tailed; bias corrected G_Hedges_ = 0.17 and 0.26, for left and right eyes, respectively) and not for Caucasian versus African faces (both eyes t(29) < 1.23, p > 0.11, one-tailed, though both tended in the hypothesized direction, bias corrected G_Hedges_ < 0.16). Additionally, the tests for the nose and mouth AOIs in the study phase yielded significant differences in three of the four comparisons (nose own- versus both other-race and mouth own-race versus African all t(29) > 2.15, p < 0.021, one-tailed, bias corrected G_Hedges_ = 0.20–0.26), where the mouth for Chinese versus Caucasian faces (t(29) > 1.33, p > 0.095, one-tailed, bias corrected G_Hedges_ = 0.17) did not reach significance.

For the test phase data (Figure C in S1 File), the same set of pairwise tests on relative frequency data from the entire period the test stimulus was visible revealed significant differences in the hypothesized direction for Caucasian versus Chinese for both eye AOIs (both t(28) > 2.89, p < 0.00038, one-tailed; bias corrected G_Hedges_ = 0.22 and 0.17, for left and right eyes, respectively) and for Caucasian versus African for the right eye (t(28) > 2.37, p < 0.013, one-tailed, bias corrected G_Hedges_ = 0.18), but not for the left (t(28) < 0.39, p > 0.35, one-tailed, bias corrected G_Hedges_ = 0.027). For the nose AOI, they revealed significantly more fixations for Chinese (t(28) > 2.78, p < 0.0048, one-tailed, bias corrected G_Hedges_ = 0.20), but not for African (t(28) < 0.60, p > 0.27, one-tailed, bias corrected G_Hedges_ = 0.034), than Caucasian faces. They also revealed significantly more fixations to the mouth for African (t(28) > 2.37, p < 0.013, one-tailed, bias corrected G_Hedges_ = 0.20), but not Chinese (t(28) < 1.14, p > 0.13, one-tailed, bias corrected G_Hedges_ = 0.11), than Caucasian faces.

Together these data show that we largely replicated in AOI analyses the prior study that reported a relatively greater proportion of fixations over both eyes for own-race faces and a relatively greater proportion of mouth and nose fixations for other-race faces.

#### Spatial Density Maps

The AOI analyses suggested a small effect of Race of face on fixation patterns. Because AOI analyses can be criticized for requiring a highly subjective *a priori* segmentation of visual features [42], we further investigated the influence of Race of face on fixation patterns through spatial density map analyses (see Methods). Because the AOI analyses suggested that the influence of Start Position on fixation patterns does not interact with Race, we pooled all Start Positions together for our spatial density map analyses to find effects of Race.

#### Spatial Density Maps: Spatial Tendencies of Fixations Differ by Race of Face

**Fig 4:**
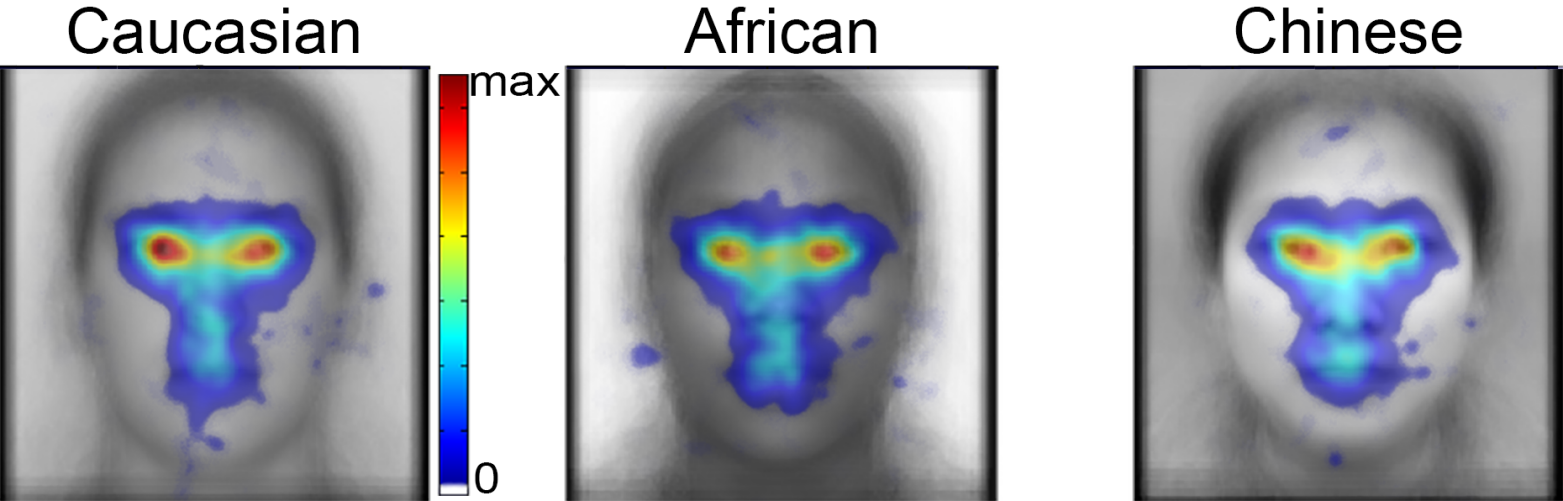
Spatial density of fixations for Caucasian, African, and Chinese faces during the study phase. The faces plotted beneath the spatial density plots are the average of all faces of the given race after alignment. Fixations are plotted as Gaussian densities summed across trials and participants. Fixation density is indicated using a color scale from zero to the maximum density value observed, with zero being transparent.

Spatial density maps on study phase data for each Race of face with all Start Positions pooled revealed fixation patterns which are consistent with the AOI relative frequency plots, but also showed more fine-grained spatial patterns in the fixations (Fig 4). The average faces for each race underlie the spatial density maps in the figure. The general shapes of the envelopes of fixation density over the faces were qualitatively similar for the different races, though some differences were apparent. Specifically, for all races, we observed the commonly reported inverted triangle shape with a strong tendency toward eyes and an overall tendency for the left side of the face. Also consistent with the AOI analyses, the spatial density maps suggest that Caucasian faces may have elicited fewer fixations to the mouth and nose regions than African and Chinese faces. Additionally, there are hints of fine-scaled spatial differences between the races in the eye and bridge regions of the faces, in addition to possibly fewer fixations to the eye-region in African faces than Caucasian and Chinese. It is also notable that Caucasian fixation densities appear to have more focal peaks than the African and Chinese faces; therefore, perhaps fixation patterns are also more diffuse for other-race faces.

#### Contrast Maps

**Fig 5:**
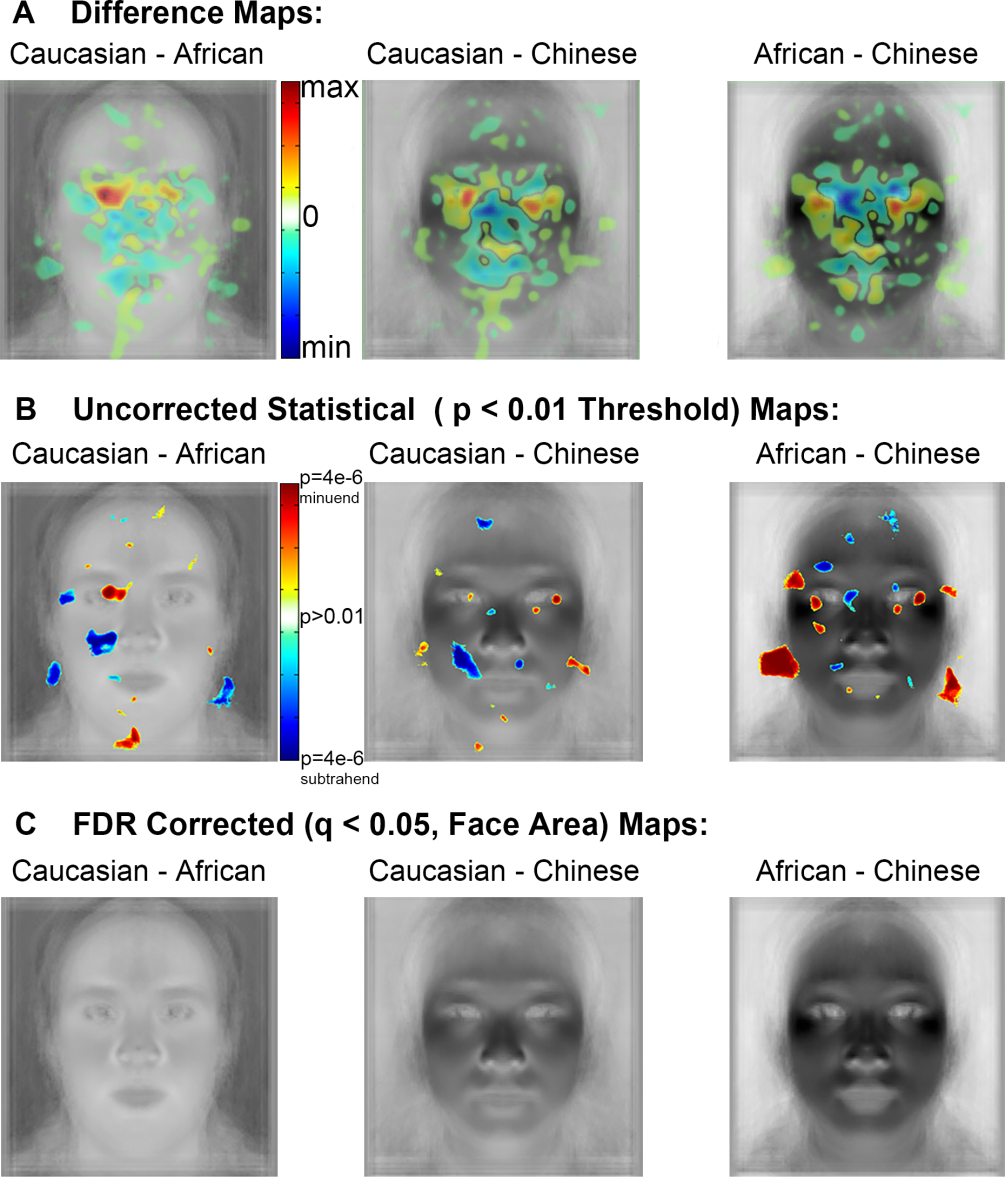
Spatial density contrast maps. (a) Spatial density difference maps showing numerically greater fixation density over the eyes and numerically lesser fixation density over the nose and mouth of own- than other-race faces. (b) Statistical maps thresholded at p < 0.01, uncorrected, suggesting fine differences in fixation density for the features of own- and other-races faces and also evidence of greater diffusivity of the spatial extent of fixation patterns for other- compared to own-race faces. (c) The same statistical maps from (b) FDR corrected at q < 0.05 reveal that no differences survive the correction.

In order to visualize the differences in fixation density suggested by the spatial density maps, we subtracted spatial density maps (Fig 5A). The underlay of these contrast maps shows the average face subtractions between races. These contrasts show that Caucasian faces elicited fewer fixations to the mouth and nose regions than African and Chinese faces. Particularly striking is the relatively higher magnitude of fixation density over the left eye of Caucasian faces compared to African and Chinese faces, in addition to fewer fixations to the eye-region in African faces than Caucasian and Chinese.

In order to determine the statistical significance of these apparent differences in fixation patterns between races of face, we produced statistical maps (see Methods). Uncorrected maps at the p < 0.01 threshold for each tail of each contrast provide evidence for more fixations to the specific parts of the eyes and fewer fixations to parts of the mouth and nose regions of own-race (Caucasian) than other-race faces (Fig 5B). However, those specific differences did not survive a correction for multiple comparisons (Fig 5C) based on FDR control (see Methods). The differences also did not survive correction when an alternative method of multiple comparisons correction, cluster size correction, was applied. Thus, while the qualitative pattern of the fixation differences between face races matches the results obtained with the AOI analyses, these differences do not reach statistical significance when correcting for the multiple comparisons that must be made across the maps.

#### Profile Analyses

Because AOI analyses can be criticized for requiring a highly subjective *a priori* segmentation of visual features [42], but spatial statistical maps can be criticized for lacking sensitivity, we conducted additional exploratory analyses that were meant to increase sensitivity without subjective segmentation. Specifically, profile plots and profile contrasts were also produced in order to detect differences in fixation patterns along individual dimensions (see Methods). The same data used for the statistical maps were collapsed along single dimensions (horizontal or vertical) to produce these profile plots and contrasts. Here we focus only on the profile plots in the y- (vertical) dimension because this dimension allows for comparing fixation densities in the eyes, nose, and mouth regions.

#### Profile Analyses: Profile Plots

**Fig 6:**
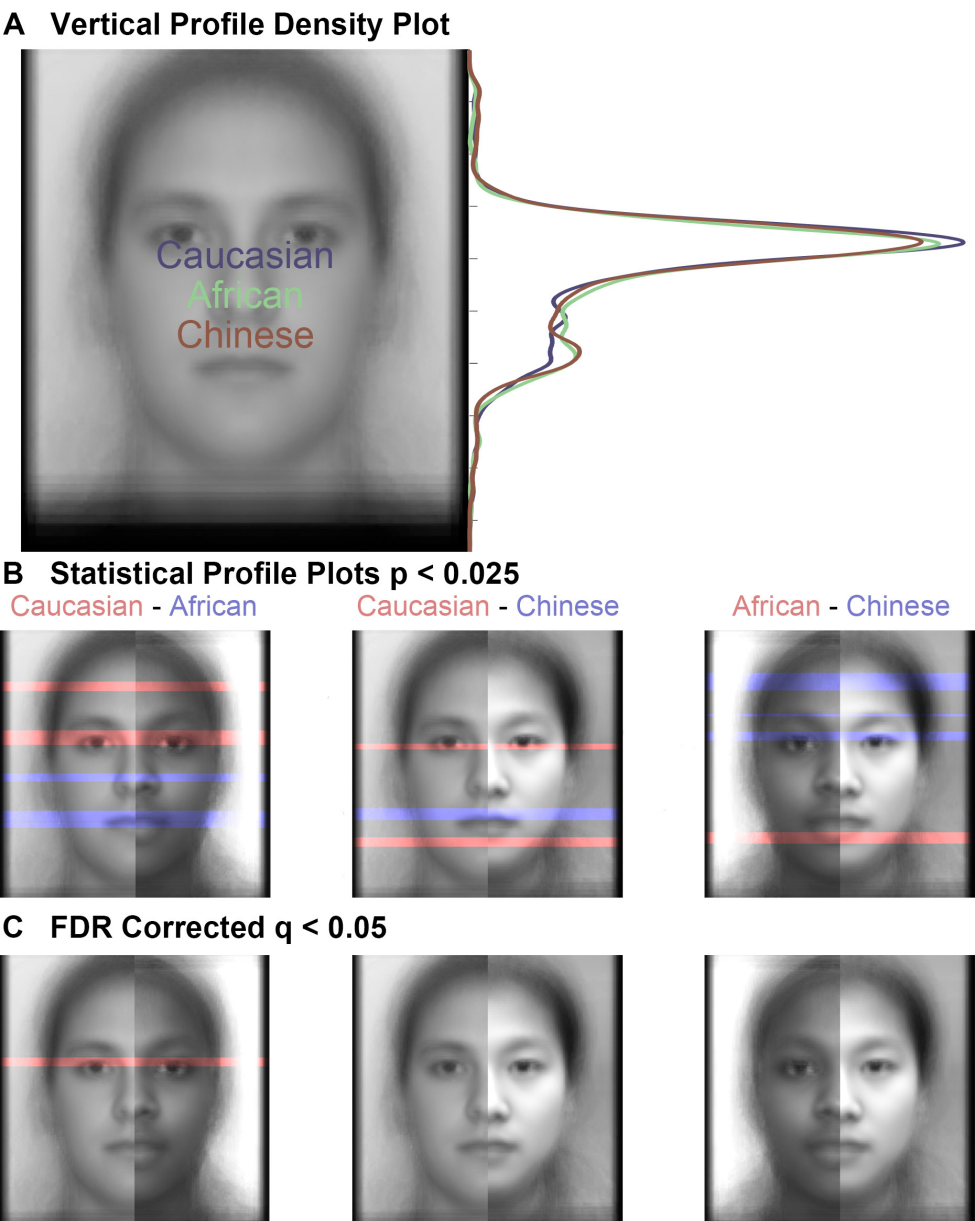
Vertical profile density plots and profile contrast plots. (a) Vertical profile densities for Caucasian, African, and Chinese faces suggesting numerically greater fixations density over the eyes and numerically lesser fixation density over the mouth for own- than other-race faces. (b) Statistical profile plots thresholded at p < 0.025, uncorrected, suggesting significantly greater fixation density over the eyes and numerically lesser fixation density over the mouth for own- than other-race faces. (c) The same statistical profile plots from (b) FDR corrected at q < 0.05 reveal that a relatively greater fixation density over the eyes for Caucasian than African faces survives the correction.

The y-profile curves again suggest a higher density of fixations in the eye regions and fewer fixations in the mouth regions for own- versus other-race faces (Fig 6A).

#### Profile Contrasts: Own-Race Eye and Other-Race Mouth and Nose Fixations

We produced profile statistical maps of the different race contrasts in order to test their significance. The threshold p-value for the uncorrected profile density maps was numerically higher than that for the uncorrected spatial density maps, in part to accommodate for the fewer multiple comparisons, but also to reveal the qualitative correspondence in eye-movement patterns between the profile density plots and the AOI analyses. These profile density maps (Fig 6B) suggest that relatively more eye-region fixations landed on Caucasian than African and Chinese faces. Also, compared to Caucasian faces, African faces had more mouth and nose fixations, and Chinese faces has more mouth fixations and fewer chin fixations. Contrasting African and Chinese faces revealed that Chinese faces received relatively more eye and forehead/hair fixations and Africans more lower lip fixations. However, when correction for multiple comparisons was applied according to FDR control (see Methods), the only significant difference remaining reflected more eye-region fixations for Caucasian than African faces (Fig 6C, Figure E in S1 File). The average effect size (unbiased G_Hedges_) of the profile density differences in the vertical pixels within this surviving significant region was G_Hedges_ = 0.64.

## Discussion

### Differences in looking at other-race faces

Two highly conflicting views have emerged from prior eye-tracking studies of the other race effect, namely (i) that the fixation pattern to faces depends on the culture of the observer, but is equivalent by race of face observed [16–25], and (ii) that the fixation pattern to faces differs by own- vs. other-race face [27–29,31], but does not differ by race of observer [26,32]. Here, we focused on the effect of race of face, not race of observer, and found evidence that fixations were indeed influenced by race of face in our Caucasian observers. Furthermore, our study provides insight into at least one reason why the discrepant reports regarding the influence of the race of face on eye-movements may exist, namely differences in the statistical sensitivity of different analysis methods employed across studies.

We investigated the effect of race of face on recognition performance and fixation patterns of Caucasian observers viewing Caucasian, African, and Chinese faces. Consistent with some previous eye-tracking studies [26–28], we found in Caucasian observers impairment for discriminating Chinese faces, less conservative criterion bias for other-race (African and Chinese) faces, and, importantly, differences in eye-movements between own- and other-race faces. Specifically, we found that for our Caucasian participants, relatively more eye-region fixations landed on Caucasian than African and Chinese faces. Also, compared to Caucasian faces, African faces received more mouth and nose fixations, and Chinese faces received relatively more nose fixations. These fixation pattern differences were, however, quite subtle. Detection of these differences was analysis dependent, suggesting that prior discrepant reports with respect to the presence of these differences may be due to differences in statistical power.

Our study is unique in that we tested participants on two other-race face categories (African and Chinese) not just one as in most prior studies. This enabled us to determine not only if recognition and fixations differ between own- and other-race faces, but also if and how they differ between different races of other-race of faces. We additionally found that our Caucasian observers were only impaired in discrimination of Chinese faces in our paradigm, though both other-race faces elicited a less stringent criterion for responding that a face had been recognized. Thus, African faces were discriminated equally as well as Caucasian faces, and this is likely due to the fact that our participants were living in the Washington DC area, which has a large African-American population.

Despite this lack of an ORE for African faces, fixation patterns differed between Caucasian and African faces. It is unclear whether this is because (i) the relative diagnostic value of different facial features differ between Caucasian and African faces and so the fixation differences reflect greater attention to those features (e.g. mouth and nose) which optimally enable individuation for each race of face [23,24], or (ii) the fixation patterns do not precisely reflect the facial information extracted [17,19,20]. Evidence for the former possibility comes from two prior eye-tracking studies [23,24] that report for Black and White observers exhibiting an ORE, that relative to White observers, Black observers tended to look more at the noses of Black and White faces, but importantly that forced nose-region fixation enabled Black faces to be recognized more accurately than White faces for both Black and White observers, whereas forced eye-region fixation enabled White faces to be recognized more accurately for both Black and White observers. Thus, the increased nose fixations to African faces in our Caucasian participants may be an explanation for their lack of an ORE (in d’) for African faces, and the increased nose fixations may be the result of a strategy acquired through our participants’ substantive experience with African faces. Evidence for the latter possibility that fixation patterns do not precisely reflect the facial information extracted comes from three eye-tracking studies that restricted information sampling of the face stimuli through virtual apertures [17,19] or virtual scotomas [20] and suggested that even though their Asian participants predominantly fixated at the center of the face, the eyes were nonetheless the region principally attended for recognition. Thus in the context of our study, it is unclear whether increased nose and mouth fixations to African faces compared to Caucasian faces indicates that those facial features were more greatly utilized for African facial recognition.

Relatedly, it is unclear how the fixation differences observed between Caucasian and Chinese faces are related to the facial information used as the differences in fixation patterns were similar to those between Caucasian and African faces, which, as just discussed, could not be related to differences in recognition performance. However, if it is the case that the diagnostic facial information tends to be in the eyes of Chinese faces, but in the nose and/or mouth of African faces, it is possible that it is precisely the same relative shift in fixation away from the eyes for both other-race faces compared to own-race faces, that lead to the presence of an ORE for Chinese faces, yet an absence of an ORE for African faces in our participants. Such a race of face difference in diagnostic facial information is consistent with the studies discussed in the preceding paragraph, though a study directly comparing between African and Chinese facial feature diagnosticity would be be needed to determine if such specific differences in facial feature diagnosticity are true. This lack of a clear relationship between eye-movements and identification performance possibly also suggests that perceptual exposure (higher to African than Asian faces in Washington, D.C.) may have less effect on eye-movements than the physiognomy associated with the race of the face [43–50]. Future studies will be needed to establish how these differential fixation patterns to different race faces relate to identification performance and processing of facial information.

### Analysis-dependent sensitivity to differences: A plausible account of prior discrepant results

Notably, however, our results reveal that these fixation differences between races of faces were rather small, and quite importantly, sensitivity for detecting these differences was analysis-dependent. Specifically, analyses utilizing Areas of Interest (AOIs) were statistically sensitive to these differences, whereas analyses of spatial densities over entire faces were not. However, spatial density analyses were qualitatively in agreement with the AOI analyses. This important result thus helps clarify one potential source of the discrepancy in the literature regarding the influence of race of face on fixation patterns, and indeed indicates an issue of broader methodical importance in the study of fixations. It is possible that because the magnitudes of these other-race effects are small that some studies have failed to detect [16–25] or only partially detected such effects [27], whereas four studies have reported similar effects [26,28,29,31] between own- and other-race faces. An additional study [32] also found differences between own- and other-race faces, and not between races of observers; however, the pattern of differences is not consistent with our results. This may be due to the specific paradigm utilized in which numbers of fixations were restricted. All of the studies that detected differences between own- and other-race faces utilized AOI analyses, whereas seven out of ten of those that did not detect any difference utilized only spatial density analyses. Two of the studies to detect a difference through AOI analysis additionally detected differences through spatial density analysis [28,29], though perhaps due to statistical inflation resulting from some limitations recently found with a portion of the analysis package that was used ([51,52]; see http://perso.unifr.ch/roberto.caldara/index.php for the updated package).

Though substantial statistical sensitivity over spatial density analysis may be increased through AOI analyses, AOI analyses have the limitation that they reduce spatial resolution and often require subjective or variable *a priori* segmentation of visual features [42]. By increased statistical sensitivity, we do not mean an increased likelihood of finding any statistically significant difference whatsoever since a spatial density map would be more likely to detect a true difference over a given region than an AOI analysis on the same data but restricted to a different region where there is no difference. Rather statistical sensitivity is greater for the AOI analysis than the spatial density analysis for a true effect within the restricted region defined by the AOI (e.g. a true difference over the left eye is more likely to be detected with a left eye AOI than a full-face spatial density analysis). The best criteria for precisely how AOIs are to be defined in a given situation are not obvious, thus creating variability in AOI definitions even for similar stimuli (e.g. faces) across studies. This variability complicates comparison of results across studies. However, recent work has indicated that, at least for face stimuli, large AOIs, especially those implemented with a Voronoi tessellation method, are the most objective, the most robust to noise, and likely the least problematic for between-group and cross-study comparison of statistics [53].

A corollary of the limitation that AOIs often require subjective *a priori* segmentation of visual features is that in the absence of precise, well-motivated *a priori* hypotheses by which to define AOIs with high specificity, the meaningfulness of effects within AOIs may be unclear or inadvertently misleading. Effects can be found only where a researcher is looking for them, such that, due to the possibility of relatively fine differences in eye-movement patterns, an effect could be detected or miss being detected should the AOIs have been defined perhaps even slightly differently. For example, a natural fixation cluster may be inadvertently artificially segmented or else, separate natural fixation clusters may inadvertently be grouped together in a way that can hide the significance of such natural clusters.

When *a priori* hypotheses that allow for quite specific definition of AOIs are absent, this limitation could be remedied by utilizing independent eye-movement spatial density data to define the AOIs in a data-driven manner. It should be noted though that, in many situations, this approach is not appropriate, such as when stimuli or data cannot be aligned. Also, even if this data-driven AOI approach has been followed across studies, it does not necessarily allow for direct comparison of the resultant AOIs across studies.

The statistical sensitivity versus resolution and meaningfulness tradeoffs between AOI and spatial density analyses are highly analogous to those respectively between Region of Interest (ROI) and whole brain analyses well known in fMRI studies (see e.g. [54,55]). It is important then to keep the same considerations in mind when analyzing and interpreting eye-movement data. Neither approach is universally more advantageous or limited than the other, though one may be more appropriate than the other in a given circumstance. For example, an AOI approach would be advantageous in detecting an effect expected to be subtle within a well defined region associated with an *a priori* hypothesis, whereas a spatial density analysis would be advantageous when attempting to map highly significant differences in a data-driven manner at high spatial resolution or when hypotheses regarding regions cannot be incontrovertibly defined specifically with an AOI. In this study we also employed profile density analyses and this approach (or a similar dimensional reduction) may be a useful technique for compromising between these tradeoffs.

### Issues requiring further investigation

In prior studies, differences between own- and other-race faces were often reported concomitantly with an absence of an effect of race of observer and vice versa. While our results potentially account for the discrepancy in prior studies in detecting effects of race of face on fixations, a limitation of our study is that it did not investigate the effects of race of observer, in which double dissociations between races of observers, characteristic of the Other-Race Effect, could be detected. The reasons behind the discrepancy in reports of the influence of race of observer on fixations to faces still requires elucidation in future studies. For example, one hypothesized cause proposed [24] as requiring investigation is a potential difference between White European/British participants and White American participants, given that many European and British research groups find that participant culture affects the way faces are viewed, whereas US teams tend to find that the ethnicity of the face is key.

The majority of the studies reporting such an influence of race of observer come from a single research group [16–22] utilizing the same base stimuli and highly similar paradigms across studies, which have all yielded patterns of fixations mainly over the center of the face for East Asian (Chinese) participants versus the “classical” T-shaped pattern with highest density over the eyes for Western Caucasian participants. A subset of those studies which have restricted information sampling of the face stimuli through virtual apertures [17,19] or virtual scotomas [20] were interpreted as revealing that though Western Caucasian and Eastern Asian participants employ different eye-movement patterns, the same facial information is attended for recognition, namely principally the eyes. It is proposed then that East Asian participants extract visual information more efficiently from extrafoveal vision and thus that the center of the face is optimal for extracting information on the eyes. A second research group comparing instead the eye-movement patterns between White and Black participants report that Black participants fixate the nose more than the eyes, and vice versa for White participants [23,24]. Interestingly, the ORE was eliminated in White participants when they were forced to initially fixate at the location preceding the nose of Black faces, and likewise when Black participants were forced to initially fixate the location preceding the region between the eyes of White faces. More specifically, nose-region fixation crosses preceding Black faces caused them to be recognized more accurately than White faces for both Black and White observers, whereas (in one [23] of the two studies from that second group) White faces were recognized more accurately by both Black and White observers when they were preceded by a eye-region fixation cross. One research group, therefore, proposes that differential eye-movements do not correspond to differential information use between races of observers, whereas the second group proposes that they do. The two groups curiously disagree conceptually over how these differences in eye-movements correspond to differences in diagnostic value of individual features between different race faces, and so clarification of this is warranted in future studies.

More fundamentally, however, the reports of a center-of-face pattern of eye-movements to faces (i.e. peak fixation density over the center of the face, rather than over the eyes) for East Asian participants at the group level (albeit with individual participant differences) [16–22] seem to be unique to the group reporting this and are thus perplexing in light of other eye-tracking studies involving East Asian participants viewing faces. In stark contrast to these reports, another eye-tracking study of the ORE [26] has reported that regardless of the race of the observer (East Asian or Caucasian), own-race faces elicited a relatively greater proportion of fixations to the eyes and hair, while other-race faces elicited a relatively greater proportion of fixations to the nose and mouth. Further, several other studies involving East Asian (Chinese or Japanese) participants viewing faces robustly show the “classical” T-shaped fixation pattern of fixations that, notably, almost exclusively demonstrate peak density over the eyes when spatial density or scatter plot analyses were utilized [28,29,32,56–59] or show greater fixation over the eyes relative to the nose when area-normalized AOI analyses were utilized [60,61]. Other studies without area normalized AOIs are also at least suggestive of the same tendency for East Asian observers to principally fixate the eyes [25,62–64]. One study with Japanese participants even found that better facial recognition performance was strongly associated with more fixations over the eyes [62], directly contradicting the account that the center of the face is the optimally informative location for East Asian observers to look for facial recognition Further, a recent study has reported that initial eye-movements to a point near the eyes during face recognition are optimal and, importantly, are similar between Caucasian and Asian observers, though not equal between races of faces[32]. The study even reports that initial fixations for Asian faces fall higher on the face (i.e. nearer to the vertical position of the pupil) than for Caucasian faces. An interesting exception to the eye-bias in fixation patterns though is a study [61] that found a nose greater than eyes (i.e. center-of-face) bias for Japanese participants viewing emotionally expressive faces, but an eyes greater than nose (i.e. “classical”) bias when viewing neutral expressions. Because emotional face stimuli were used in the ORE studies reporting a center-of-face fixation bias for East Asian, but not for Western Caucasian, participants [16–22], it would be valuable to investigate whether the use of emotional stimuli could be a factor contributing to this perplexing differential pattern (though see [65]).

## Conclusion

Caucasian observers in our study exhibited impairment for discriminating Chinese faces, less conservative criterion bias for other-race (African and Chinese) faces, and, importantly, differences in eye-movements between own- and other-race faces. Relatively more eye-region fixations landed on Caucasian than African and Chinese faces. Also, relative to Caucasian faces, African faces received more mouth and nose fixations, and Chinese faces received relatively more nose fixations. These were very subtle fixation pattern differences, however, and detection of these differences was analysis dependent. Thus, prior discrepant reports with respect to the presence of these differences are potentially due to differences in the statistical power of different analytical approaches across studies. Further study is warranted now to account for another discrepancy in prior reports regarding the presence and meaning of differences in eye-movement patterns to faces between different racial groups of observers.

## Supporting Information

S1 File Supplemental Material. (DOCX)
