# Supplementary material for: Differences in Looking at Own- and Other-Race Faces Are Subtle and Analysis-Dependent: An Account of Discrepant Reports
Source: PLoS One. 2016 Feb 5;11(2):e0148253. doi: 10.1371/journal.pone.0148253 (PMC4744017; doi:10.1371/journal.pone.0148253)
Supplement: S1 File — (DOCX) [file pone.0148253.s001.docx]

**Supplemental Material**

**Temporal Dynamics of Fixations (Supplemental)**

To examine fixation patterns, we first focused on the temporal duration of individual fixations. A three-way ANOVA on study phase fixation durations with Ordinal Fixation (1^st^ through 5^th^), Race (Caucasian, African, Chinese) and Start Position (left, right, up, down) as within-subject factors revealed a significant main effect of Ordinal Fixation (F(4,116) > 23.67, p < 0.001, Greenhouse-Geisser corrected), and Start Position (F(3,87) > 3.47, p < 0.030, Greenhouse-Geisser corrected), but not of Race (F(2,58) < 1.25, p > 0.28, Greenhouse-Geisser corrected). Qualitatively, there was a tendency for longer fixations for Chinese faces at the later ordinal fixations, although the interaction of Ordinal Fixation and Race (F(8,232) > 2.10, p < 0.083, Greenhouse-Geisser corrected) was not significant. All other interactions were not significant (all p > 0.33, Greenhouse-Geisser corrected). Thus, in the first five fixations of the study phase while both Start Position and Ordinal Fixation affected fixation durations independently, there is no evidence that Race did.

**Figure A. Fixation durations of each of the first five fixations during the study phase.** The first fixation was significantly shorter than the subsequent fixations, as was the second fixation. Error bars indicate between-subject standard errors.

A similar three-way ANOVA on test phase fixation durations with Ordinal Fixation (1^st^ and 2^nd^ only, due to short stimulus presentations in the test phase truncating many fixation durations beyond the 2^nd^ fixation), Race (Caucasian, African, Chinese) and Start Position (left, right, up, down) as within-subject factors revealed a significant main effect of Ordinal Fixation (F(1,28) > 52.38, p < 0.001, Greenhouse-Geisser corrected, η_p_^2^ = 0.65), and a marginal main effect of Race (F(2,56) > 2.88, p < 0.073, Greenhouse-Geisser corrected, η_p_^2^ = 0.094), but not of Start Position (F(3,84) < 1.50, p > 0.22, Greenhouse-Geisser corrected, η_p_^2^ = 0.051). All interactions were not significant (all p > 0.25, Greenhouse-Geisser corrected, η_p_^2^ < 0.047). Thus only Ordinal Fixation significantly affected fixation durations in the first two fixations of the test phase.

**Figure B. Fixation durations for each of the first two fixations during the test phase.** The first fixation was significantly shorter than the second fixation. Error bars indicate between-subject standard errors.

Prior studies have indicated that facial information for individuation is not deeply processed during the first fixation to a face. In particular, Hsiao and Cottrell (2008) found that at least two fixations are needed for optimal face recognition performance. Further, in our earlier study [32], we found that the first fixation was significantly shorter than the subsequent fixations. To determine if the same held true for the current data we conducted follow-up pairwise comparisons on Ordinal Fixation (Figure S1) first on the study phase, demonstrating that average duration of the first fixation was significantly shorter than subsequent (2^nd^ through 5^th^) fixations (all four comparisons t(29) > 6.31, p < 0.001, one tailed). Further, the second fixation was also significantly shorter than subsequent (3^rd^ through 5^th^) fixations (all three comparisons t(29) > 8.46, p < 0.009, two-tailed), but no other comparisons yielded significant differences (all three t(29) < 0.77, p > 0.44, two-tailed).

Comparison of the first and second fixations in the test phase (Figure S2), also demonstrated that average duration of the first fixation was significantly shorter than the second (t(28) > 7.22, p < 0.001, one tailed). Given the findings of Hsiao and Cottrell and following our earlier work, our subsequent analyses were primarily conducted with the first fixation removed, although qualitatively the same results were observed when it was included (see *Supplementary Materials: Fixation Patterns*).

We additionally investigated the influence of Phase (Study, Test) on the temporal dynamics of fixations. A two-way ANOVA on subject average fixation durations with Ordinal Fixation (1^st^ and 2^nd^) and Phase (Study, Test) as within-subject factors revealed main effects of Ordinal Fixation (F(1,28) > 77.64, p < 0.001, η_p_^2^ = 0.74) and of Phase(F(1,28) > 19.41 p < 0.001, η_p_^2^ = 0.41), as well as an interaction between Ordinal Fixation and Phase (F(1,28) > 8.70, p < 0.007, η_p_^2^ = 0.24) indicating that the temporal dynamics of fixations differed between Study and Test Phase though the first fixation was significantly shorter in duration than the second fixation for both phases. Indeed, post-hoc paired comparisons between Study and Test Phase revealed significantly longer duration fixations for Test Phase for both the first (t(28) < -2.11, p < 0.045, two-tailed uncorrected) and second (t(28) < -4.22, p < 0.001, two-tailed uncorrected) fixations. This influence of Phase seen in our data may reflect the difference between encoding and recognizing or it may reflect the influence of restricted time to view the stimulus (i.e. one second limit in the test phase).

**Differences in Fixation Patterns for Study versus Test Phases by Ordinal Fixation**

Because it has been shown that not all ordinal fixations are equally important in processing facial information and also that the first two fixations suffice for face recognition [40], we analyzed fixation patterns across AOIs in more detail by considering the first three fixations separately. Further, because subjects were implicitly performing different tasks on faces between the Study and Test phases, we additionally investigated the influence of Phase on fixation patterns for each of the first three fixations (Figure S4).

**Figure C. Distribution of fixations across AOIs for own- and other-race faces during the test phase.** (a) Relative frequencies of fixations for each race of face across AOIs for the all fixations except the first pooled across the entire period during which the stimulus was visible. Error bars indicate between-subject standard errors. (b) Within-subject differences among race of face conditions from (a) reveal significantly more eye fixations for Caucasian than Chinese faces, significantly more right eye fixations for Caucasian than African faces, and significantly fewer mouth fixations for Caucasian than African faces. Error bars indicate the within-subject standard error.

**Figure D. Study (a) versus test (b) phase distributions across AOIs for each of the first three fixations.** Relative to study phase, there were significantly fewer fixations to the left eye and significantly more fixations to the nose in the test phase for the second and third fixations. Error bars indicate between-subject standard errors.

For the first fixation, a three-way ANOVA on the relative frequency of fixations with AOI (left eye, bridge, right eye, nose, and mouth), Race (Caucasian, African, Chinese), and Phase (study, test) as within-subject factors yielded a main effect of AOI (F(4,112) > 27.72, p < 0.001, Greenhouse-Geisser corrected, η_p_^2^ = 0.50) and marginal main effect of Race (F(2,56) > 3.13 , p < 0.059, Greenhouse-Geisser corrected, η_p_^2^ = 0.10), but no main effect of Phase (F(1,28) < 0.002, p > 0.97, Greenhouse-Geisser corrected, η_p_^2^ = 0). The interaction of AOI and Race (F(8,224) > 4.318, p< 0.005, Greenhouse-Geisser corrected, η_p_^2^ = 0.13) was significant, but no other interactions were (all p > 0.42, Greenhouse-Geisser corrected, η_p_^2^ < 0.032). These indicate that for the first fixation, our participants employed different fixation patterns for different races, but did not seem to employ different patterns between Study and Test Phase. Prior studies have indicated that facial information for individuation is not deeply processed during the first fixation [32,40] and our prior analyses showed that the first fixation was distinct from the later fixations in terms of fixation duration, so our interest focused on subsequent fixations.

For the second fixation, the same three-way ANOVA yielded main effects of AOI (F(4,112) > 3.62, p < 0.021, Greenhouse-Geisser corrected, η_p_^2^ = 0.12) and Race (F(2,56) > 5.97 , p< 0.012, Greenhouse-Geisser corrected, η_p_^2^ = 0.18), but no main effect of Phase (F(1,28) < 0.97, p > 0.33, Greenhouse-Geisser corrected, η_p_^2^ = 0.033). The interaction of AOI and Race (F(8,224) > 2.78, p < 0.013, Greenhouse-Geisser corrected, η_p_^2^ = 0.090) was again significant, and notably the interaction of AOI and Phase (F(4,112) > 9.63, p< 0.001, Greenhouse-Geisser corrected, η_p_^2^ = 0.26) was also significant, reflecting relatively more left eye fixations for study and relatively more nose fixations for the test phase (see below). The other interactions were not significant (both p > 0.44, Greenhouse-Geisser corrected, η_p_^2^ < 0.034). These indicate that in the second fixation, Race and Phase both influenced fixation patterns, but seem to have done so independently.

For the third fixation, a final three-way ANOVA yielded the same main effects and interactions as the second fixation did. Main effects of AOI (F(4,112) > 6.52, p< 0.002, Greenhouse-Geisser corrected, η_p_^2^ = 0.19) and Race (F(2,56) > 3.46 , p< 0.039, Greenhouse-Geisser corrected, η_p_^2^ = 0.11) were again significant, but not the main effect of Phase (F(1,28) < 1.50 p > 0.23, Greenhouse-Geisser corrected, η_p_^2^ = 0.051). The interaction of AOI and Race (F(8,224) > 3.101, p < 0.011 Greenhouse-Geisser corrected, η_p_^2^ = 0.10) and of AOI and Phase (F(4,112) > 5.74, p < 0.002, Greenhouse-Geisser corrected, η_p_^2^ = 0.17) were also significant, but no other interactions (both p > 0.31, Greenhouse-Geisser corrected, η_p_^2^ < 0.041). These indicate that in the third fixation, like in the second fixation, Race and Phase both influenced fixation patterns independently. Further ordinal fixations could not be reliably compared individually or in aggregate between Study and Test phases given the limited duration (1 second) of the presentation of the stimuli in the test phase.

We found influences of Phase (Study, Test) and Race (Caucasian, African, Chinese) on fixation patterns for the second and also for the third ordinal fixations. For this reason, we more closely investigated the influence of Phase. In our data for second and third fixations, post-hoc paired test phase versus study phase comparisons on relative frequency of fixation at each AOI when Race conditions were pooled together yielded several significant differences. For the second fixation, the left eye AOI contained significantly more fixations (t(28) > 4.31, p < 0.001, two-tailed uncorrected) for the study than test phase, and the nose AOI contained significantly fewer fixations (t(28) < -3.57, p < 0.002, two-tailed uncorrected) for the study than test phase. All other AOIs yielded no significant differences (all t(28) < |1.57|, p > 0.12, two-tailed uncorrected) for the second fixation. For the third fixation, the same pattern of differences emerged. The left eye AOI contained significantly more fixations (t(28) > 3.55, p < 0.002, two-tailed uncorrected) for the study than test phase, the nose AOI contained significantly fewer fixations (t(28) < -4.27, p < 0.001, two-tailed uncorrected) for the study than test phase, and all other AOIs yielded no significant differences (all t(28) < |1.28|, p > 0.21, two-tailed uncorrected) for the third fixation. It is not clear, however, if these differences reflect encoding versus recognizing or rather a modification of eye-movement strategy due to the short duration (limit of one second) of the stimuli in the test phase. For this reason, our subsequent analyses focus just on data from the study phase, where viewing time was under the control of the participant. The potential influence of encoding versus recognizing a face on fixation patterns and visual processing warrants deeper investigation in future experiments.

**A note about the Study and Test phase eye-movement differences**

A point worth further investigation, with potentially broad theoretical or methodological implications, is the modulation of fixation patterns between study and test phase we observed in the current study. We observed significantly more fixations to the left eye, and also significantly fewer fixations to nose AOI, for the study than test phase for both the second and third ordinal fixations. Longer duration fixations were also observed in the test compared to the study phase. Other studies have reported modulations of fixation patterns between study and test phase (e.g. Henderson et al., 2005; Hsiao & Cottrell, 2008), but like in our study those also had time restricted stimuli or speeded response in the test phases. Like those studies, in the context of our paradigm it is not clear whether the differences we observe reflect the difference in implicit task (encoding versus recognition) being performed between the two phases, or rather that fixation dynamics were modified because time to view the stimulus was limited (up to 1 second) in the test phase. These two possibilities are not mutually exclusive, but if the former drives this phenomenon, it may indicate an important set of mechanisms in visual perception, and if driven by the latter, it would suggest that caution should be applied in designing and interpreting eye-movement studies with speeded response or restricted time tasks.

**Fixation Patterns: AOIs (Supplemental all 5 fixations)**

*Independent Influences of Race of Face and Start Position*

Area of Interest (AOI) analyses revealed that Race and Start Position both influence fixation patterns, but further that their influences are independent. A three-way ANOVA on the relative frequency of fixations in the study phase with AOI (left eye, bridge, right eye, nose, and mouth), Race (Caucasian, African, Chinese) and Start Position (left, right, up, down) as within-subject factors, on the relative frequency of fixations revealed a significant main effect of AOI (F(4,116) > 5.40, p < 0.004, Greenhouse-Geisser corrected, η_p_^2^ = 0.16), which indicates that not all AOIs were fixated with equal frequency. Further, there was a significant interaction between Race and AOI (F(8, 232) > 3.05, p < 0.008, Greenhouse-Geisser corrected, η_p_^2^ = 0.095) indicating that fixation patterns varied by Race. Finally, there was also a significant interaction between Start Position and AOI (F(12, 348) > 9.32, p < 0.001, Greenhouse-Geisser corrected, η_p_^2^ = 0.24) indicating that fixation patterns varied by Start Position. There was no significant three-way interaction among AOI, Race, and Start Position (F(24, 696) < 1.26, p > 0.24, Greenhouse-Geisser corrected, η_p_^2^ = 0.041), suggesting that the influences of Race and Start Position on fixation pattern are independent.

*Small but Systematic Influences of Race of Face*

The preceding analysis suggested an effect of Race of face on fixation patterns. To test for replication of a prior study [26] specifically reporting a greater proportion of fixations over both eyes in own-race faces and greater proportion of mouth and nose fixations for other-race faces, we conducted separate one-way ANOVAs on the study phase, with Race as the within-subject factor, on left eye, right eye, nose and mouth AOIs and performed the relevant pairwise comparisons between races for each of these AOIs. For both eye AOIs, only marginal main effects of Race were found (left eye: F(2,58) < 2.89 p > 0.068, Greenhouse-Geisser corrected, η_p_^2^ = 0.090; right eye: F(2,58) < 2.70, p > 0.075, Greenhouse-Geisser corrected, η_p_^2^ = 0.085). However, additional planned pairwise comparisons testing the hypothesis of greater proportion of fixations for own- (Caucasian) versus other-race (African or Chinese) in these AOIs revealed significant differences in the hypothesized direction for three of the four contrasts (all three t(29) > 1.81, p < 0.04, one-tailed, G_Hedges_ > 0.17). Only the Caucasian versus African contrast in the right eye AOI failed to reach significance (t(29) < 0.94, p > 0.17, one-tailed, G_Hedges_ = 0.11), though it tended in the hypothesized direction. Similar one-way ANOVAs for the mouth and nose AOIs revealed a significant main effect of Race in the mouth AOI (F(2,58) > 3.47, p < 0.042, Greenhouse-Geisser corrected, η_p_^2^ = 0.11) but only a marginally significant main effect of Race (F(2,58) < 2.76, p < 0.075, Greenhouse-Geisser corrected, η_p_^2^ = 0.087) in the nose AOI. The pairwise comparisons, now testing the hypothesis of greater proportion of fixations for other- (African and Chinese) versus own-race (Caucasian) in the mouth and nose AOIs revealed that all four comparisons again tended in the hypothesized direction, but only African versus Caucasian comparisons were significant for mouth and nose AOIs (both t(29) > 2.60, p < 0.008, one-tailed, G_Hedges_ > 0.22). Though the Chinese versus Caucasian comparisons for mouth and nose failed to reach significance, both were marginally significant (both t(29) > 1.46, p < 0.077, one-tailed, G_Hedges_ < 0.15). A plot of the relative frequencies of fixations falling in our AOIs for each race (Figure 2) highly resembles the analogous plot in the prior study (Figure 2 in Goldinger, He, & Papesh, 2009, which is the data for Caucasian observers looking at Caucasian and Asian faces with five second encoding duration), even though the precise way our AOIs were drawn differs slightly and our data utilizes only the first five fixations. Notably, as previously indicated, relative frequency differences between own- and other-race faces in the left eye, right eye, nose, and mouth AOIs all tended in the same direction as the prior study.

**q values within the significant region of the vertical profile density contrasts**

**Figure E. Plot of q-values within the region of significant differences in the vertical profile contrast plots.** This is a plot of the q-values within the eye region from Fig 6c showing relatively more vertical profile fixations landing on Caucasian than African faces. In this plot, the y-axis is the independent variable (pixel within the vertical region) so that the orientation of the plot allows for direct comparison with Fig 6c.

**Analyses of AOI size variability across race of face**

The following analyses of AOI areas, widths, and heights provide rough indices of race of face physiognomic differences and variability.

*AOI areas*

**Figure F. AOI areas.** Mean AOI areas. Error bars represent standard deviations.

Independent samples t-tests revealed that area within the bridge AOI was significantly greater for Chinese compared to Caucasian faces (t(37.46) = 3.71, p < 0.00075, uncorrected), the areas within the nose AOI were significantly greater for African (t(31.27) = 2.19, p < 0.036, uncorrected) and Chinese (t(26.54) = 3.58, p < 0.0011, uncorrected) compared to Caucasian faces, and the area within the mouth AOI was significantly greater for African compared to Caucasian (t(37.98) = 5.37, p < 0.00005, uncorrected) and Chinese faces (t(34.92) = 5.76, p < 0.00005, uncorrected).

*AOI widths*

**Figure G. AOI widths.** Mean AOI areas. Error bars represent standard deviations.

Independent samples t-tests revealed that widths of the bridge AOI were significantly greater for African (t(37.53) = 2.24, p < 0.040, uncorrected) and Chinese (t(37.07) = 3.68, p < 0.0007, uncorrected) compared to Caucasian faces, the widths of the nose AOI significantly differed among all races (all t > 2.84, p < 0.0077, uncorrected), and the width of the mouth AOI was significantly greater for African compared to Caucasian (t(37.89) = 3.54, p < 0.0012, uncorrected) and Chinese faces (t(37.99) = 2.69, p < 0.011, uncorrected).

*AOI heights*

**Figure H. AOI heights.** Mean AOI areas. Error bars represent standard deviations.

Independent samples t-tests revealed that height of the nose AOI was significantly less for African compared to Chinese faces (t(36.50) = 2.56, p < 0.015, uncorrected) and the height of the mouth AOI was significantly greater for African compared to Caucasian (t(37.75) = 4.72, p < 0.00005, uncorrected) and Chinese faces (t(38.00) = 5.00, p < 0.00005, uncorrected).
